# Antihypertensive Effects of Lotus Seed (*Nelumbo nucifera* Gaertn.) Extract via eNOS Upregulation and Oxidative Stress Reduction in L-NAME-Induced Hypertensive Rats

**DOI:** 10.3390/ph18081156

**Published:** 2025-08-04

**Authors:** Anjaree Inchan, Tippaporn Bualeong, Worasak Kaewkong, Nitra Nuengchamnong, Phapada Apaikawee, Pakaporn Sa-Nguanpong, Wiriyaporn Sumsakul, Natthawut Charoenphon, Usana Chatturong, Watcharakorn Deetud, Krongkarn Chootip

**Affiliations:** 1 Faculty of Medicine, Praboromarajchanok Institute, Ministry of Public Health, Nonthaburi 11000, Thailand; anjaree.inc@pi.ac.th; 2 Department of Physiology, Faculty of Medical Science, Center of Excellence for Innovation in Chemistry, Naresuan University, Phitsanulok 65000, Thailand; tippapornb@gmail.com (T.B.); phapadaa64@nu.ac.th (P.A.); pakapornsa65@nu.ac.th (P.S.-N.); chatturong.u@gmail.com (U.C.); watcharakornd59@gmail.com (W.D.); 3 Department of Biochemistry, Faculty of Medical Sciences, Naresuan University, Phitsanulok 65000, Thailand; worasak_bc@hotmail.com; 4 Science Laboratory Centre, Faculty of Science, Naresuan University, Phitsanulok 65000, Thailand; nitran@nu.ac.th; 5 Expert Center of Innovative Herbal Products, Thailand Institute of Scientific and Technological Research, Pathum Thani 12120, Thailand; wiriyaporn@tistr.or.th; 6 Department of Anatomy, Faculty of Medical Science, Naresuan University, Phitsanulok 65000, Thailand; natthawutch@nu.ac.th

**Keywords:** *Nelumbo nucifera* Gaertn., lotus seed, hypertension, blood pressure, angiotensin II

## Abstract

**Background/Objectives:** *Nelumbo nucifera* Gaertn. (lotus) seeds have traditionally been used to treat hypertension, though their mechanisms remain unclear. This study investigated the antihypertensive effects of lotus seed extract (LSE) and its mechanisms in rats with N^ω^-nitro-L-arginine methyl ester (L-NAME)-induced hypertension. **Methods:** Male Sprague Dawley rats received L-NAME (40 mg/kg/day) in drinking water and were treated orally with LSE (5, 10, or 100 mg/kg/day), captopril (5 mg/kg/day), or a combination of LSE and captopril (2.5 mg/kg/day each) for 5 weeks. Hemodynamic parameters and histological changes in the left ventricle and aorta were assessed. Mechanistic studies included measurements of plasma nitric oxide (NO) metabolites, malondialdehyde (MDA), superoxide dismutase (SOD) activity, angiotensin II (Ang II), angiotensin-converting enzyme (ACE) activity, and protein expression via western blot. **Results:** L-NAME elevated systolic blood pressure and induced cardiovascular remodeling, oxidative stress, and renin-angiotensin system activation. LSE treatment reduced blood pressure, improved antioxidant status, increased NO bioavailability, and downregulated gp91^phox^ and AT_1_R expression. The combination of low-dose LSE and captopril produced stronger effects than LSE alone, with efficacy comparable to captopril. **Conclusions:** These findings suggest that LSE exerts antihypertensive effects via antioxidant activity and inhibition of the renin-angiotensin system, supporting its potential as an adjunct therapy for hypertension.

## 1. Introduction

Hypertension is a major risk factor contributing to cardiovascular disease, which is a leading cause of global morbidity and mortality [1]. The development of hypertension is often attributed to an imbalance of vasoactive agents produced by the vascular endothelium, along with the overactivity of the sympathetic nerve-innervated vasculature [2]. Nitric oxide (NO) is a crucial vasodilator released from the vascular endothelium, plays a key role in regulating vascular tone. NO is synthesized endogenously from the amino acid L-arginine by the enzyme endothelial NO synthase (eNOS) [3]. Inhibition of NO release by N^ω^-nitro-L-arginine methyl ester (L-NAME), a non-specific inhibitor of eNOS, induces vasoconstriction and elevated blood pressure (BP). Long-term treatment with L-NAME has been reported to cause sustained hypertension, cardiovascular dysfunction, and remodeling in animal models [4]. In L-NAME-induced hypertensive rats, increased reactive oxygen species (ROS) production and reduction in antioxidant defense systems have been observed [5]. Previous studies have shown that in NO-deficient hypertensive rats, elevated superoxide (O_2_^−^) production and the increased levels of malondialdehyde (MDA) are mediated by the upregulation of the nicotinamide adenine dinucleotide phosphate (NADPH) oxidase subunit 2 (gp91^phox^) [5,6]. Furthermore, oxidative stress in rats with L-NAME-induced hypertension has been associated with the activation of the renin-angiotensin system (RAS) [6,7].

The RAS plays a critical role in the development of hypertension, primarily through angiotensin II (Ang II), which acts as a potent vasoconstrictor and enhances sympathetic nerve activity [8]. Ang II also promotes the production of ROS via AT_1_R activation, contributing to increased vascular wall thickness [9,10]. Previous studies have demonstrated that in rats treated with L-NAME, Ang II stimulates vascular superoxide (O_2_^−^) production, which impairs endothelial function and contributes to hypertension [6,7].

Captopril, a widely used angiotensin-converting enzyme (ACE) inhibitor for the treatment of hypertension, was employed as a positive control in this study. It has been shown to reduce MDA levels and enhance antioxidant activity in hypertensive rats [7,10].

However, captopril is associated with side effects such as rash, cough, loss of appetite, and angioedema [11]. Therefore, this study aims to explore new and effective herbal medicines with the potential to alleviate hypertension.

*Nelumbo nucifera* Gaertner, commonly known as lotus, is an aquatic perennial plant from the Nymphaeaceae family. It has been traditionally used in Asia to treat skin diseases, spermatorrhea, hemoptysis [12], and hypertension [13]. Extracts from various parts of the lotus plant, including rhizomes, leaves, flowers, and seeds, have been reported to provide numerous health benefits [14,15]. Lotus seeds, in particular, have shown diverse therapeutic potentials, as demonstrated in studies highlighting their anti-inflammatory [16], antioxidant [17], hepatic and renal-protective effects [18], antihypertensive [19], and immunomodulatory effects [20]. They are rich in essential nutrients, including proteins, carbohydrates, lipids, vitamins, and minerals, and also contain bioactive compounds such as flavonoids, phenolic compounds, and alkaloids [21]. A previous study demonstrated that neferine, an alkaloid from lotus seed embryos, exhibits an antihypertensive effect by inducing vasorelaxation in isolated aorta through the eNOS/NO/sGC pathway and calcium antagonism [22]. Neferine has also been shown to lower BP and mitigate vascular remodelling in spontaneously hypertensive rats [19]. Similarly, our previous study showed that administering 10 mg/kg of lotus seed extract effectively reduces BP and improves reproductive dysfunction in male rats with L-NAME-induced hypertension [23]. Despite these promising findings, information on the antihypertensive effects of lotus seed extract (LSE) and its underlying mechanisms remains limited. This study aimed to investigate the effects of LSE on BP, cardiovascular alterations, and the potential mechanisms in L-NAME-induced hypertensive rats.

## 2. Results

### 2.1. Chemical Composition of LSE by LC-MS

**Figure 1:**
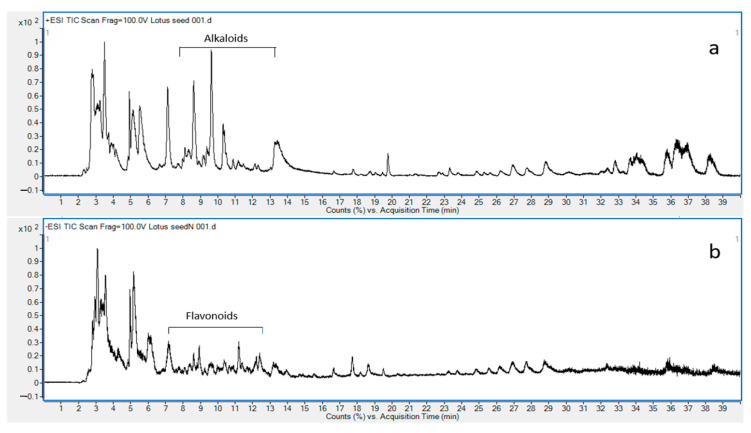
Total ion Chromatogram (TIC) of chemical constituents of LSE at concentration 20 mg/mL (**a**) positive mode, (**b**) negative mode.

LC-ESI-QTOF-MS/MS has been a widely used analytical technique for the structural elucidation of natural compounds. The accurate mass data and fragmentation pattern of each compound are unique and can be used to profile chemical constituents in crude extract, which is essential for quality control to ensure safety and efficacy. The total ion chromatogram of LSE performed in both positive mode and negative mode is shown in Figure 1. The proposed alkaloids and flavonoids are demonstrated in Supplementary Table S1.

### 2.2. Effect of LSE on Systolic Blood Pressure and Hemodynamic Parameters in L-NAME Rats

**Figure 2:**
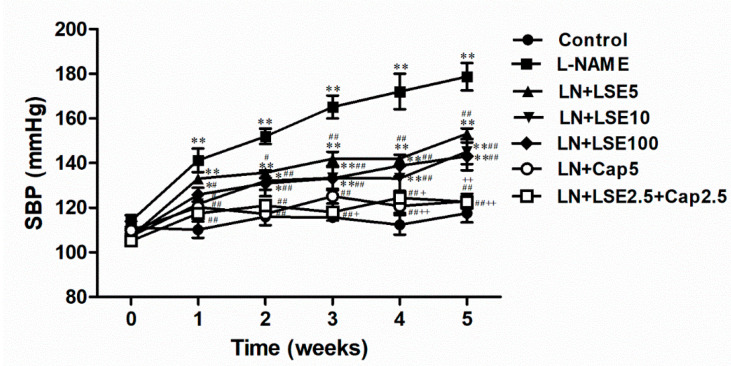
Effects of LSE and captopril on systolic blood pressure in L-NAME-induced hypertensive rats. The control group received distilled water, while hypertensive groups were given L-NAME (40 mg/kg/day) in drinking water and treated with distilled water, LSE (5, 10, 100 mg/kg/day), captopril (5 mg/kg/day), or both (2.5 mg/kg each) for five weeks. The data is presented as mean + SEM (*n* = 8). * *p* < 0.05, ** *p* < 0.01 versus control, ^#^ *p* < 0.05, ^##^ *p* < 0.01 versus L-NAME, ^+^ *p* < 0.05, ^++^ *p* < 0.01 versus LSE 100. CAP, captopril; L-NAME, LN, N^ω^-nitro-L-arginine methyl ester; LSE, lotus seed extract; SBP, systolic blood pressure.

At week 0, SBP and HR measured in conscious rats using tail-cuff plethysmography method showed no significant differences among groups (Figure 2). After five weeks of L-NAME treatment, SBP progressively increased to 178.72 ± 6.17 mmHg, significantly higher than in the control group (117.50 ± 4.01 mmHg, *p* < 0.01, Figure 2). LSE (5, 10, and 100 mg/kg) reduced SBP in hypertensive rats (*p* < 0.01 vs. L-NAME). Treatment with captopril (5 mg/kg) or a combination of LSE (2.5 mg/kg) and captopril (2.5 mg/kg) effectively normalized SBP to control level (122.94 ± 3.27 and 122.62 ± 2.01 mmHg, Figure 2). At week 5, direct measurement of hemodynamic parameters (SBP, DBP, MAP, and HR) via the common carotid artery in anesthetized rats confirmed that L-NAME successfully induced hypertension, as evidenced by significant increases in all parameters (*p* < 0.05, 0.01 vs. normotensive control, Table 1). Administration of LSE (5, 10, and 100 mg/kg) significantly reduced these values (*p* < 0.05, 0.01 vs. L-NAME group), while captopril (5 mg/kg) alone or a combination of LSE (2.5 mg/kg) and captopril (2.5 mg/kg) fully restored them to normotensive level (Table 1).

### 2.3. Effect of the LSE on Body Weight and Organ Weights in L-NAME Rats

At baseline (week 0), there were no significant differences in body weight among the experimental groups. After 5 weeks of treatment, body weight and the relative weights of the heart and liver did not differ significantly among the groups (Table 1).

### 2.4. Effect of LSE on Left Ventricular (LV) Morphometry in L-NAME Rats

**Figure 3:**
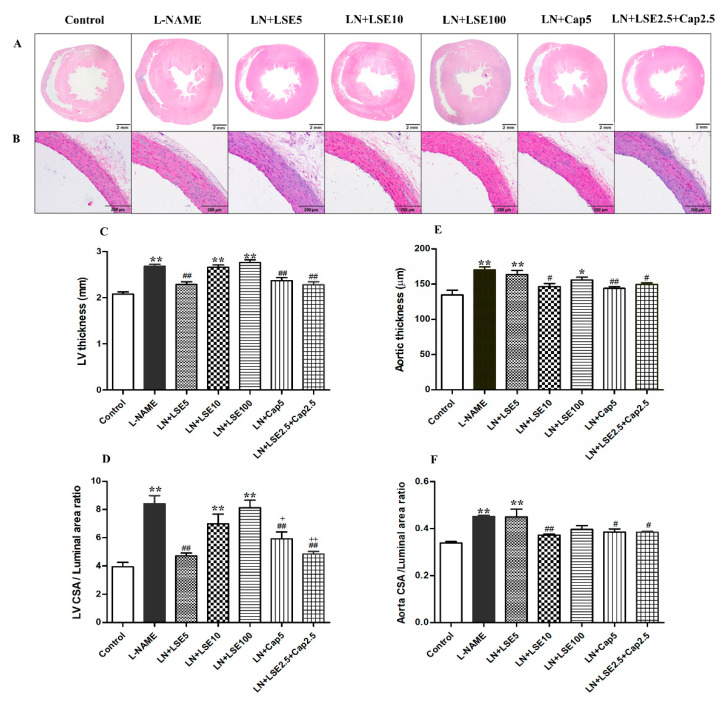
Effects of LSE and captopril on cardiovascular morphology in L-NAME-induced hypertensive rats. Representative hematoxylin and eosin-stained section observed under a light microscope: (**A**) Cross-section of the rat heart at 1.25× magnification (scale bar = 2 mm), and (**B**) cross-section of the abdominal aorta at 20× magnification (scale bar = 200 µm). Quantitative analyses of cardiovascular remodeling are represented as (**C**) left ventricular (LV) wall thicknesses, (**D**) LV cross-section area to luminal area ratio, (**E**) abdominal aortic wall thickness, and (**F**) aortic cross-section area to luminal area ratio. Values are expressed as mean ± SEM (*n* = 5–6). * *p* < 0.05, ** *p* < 0.01 vs. control, ^#^ *p* < 0.05, ^##^ *p* < 0.01 vs. L-NAME, ^+^ *p* < 0.05, ^++^ *p* < 0.01 versus LSE 100. CAP, captopril; L-NAME or LN, N^ω^-nitro-L-arginine methyl ester; LSE, lotus seed extract; LV, left ventricular.

L-NAME-induced hypertensive rats showed increased LV wall thickness (2.68 ± 0.05 vs. 2.08 ± 0.05 mm) and LV cross-section area/luminal area ratio (8.41 ± 0.59 vs. 3.94 ± 0.32) compared to control (*p* < 0.01, Figure 3A,C,D). These changes were significantly attenuated by treatment with LSE (5 mg/kg), captopril (5 mg/kg), or their combination (2.5 mg/kg each) (*p* < 0.01 vs. L-NAME). Higher LSE doses (10 and 100 mg/kg) had no effect (Figure 3A,C,D).

### 2.5. Effect of LSE on Abdominal Aortic Wall Thickness in L-NAME Rats

L-NAME-induced hypertensive rats showed increased aortic wall thickness compared to control (167.00 ± 4.28 vs. 135.92 ± 5.77 µm, *p* < 0.01, Figure 3B,E,F). These changes were significantly attenuated by treatment with LSE (10 mg/kg), captopril (5 mg/kg), or their combination (2.5 mg/kg each) (*p* < 0.05, 0.01 vs. L-NAME). LSE (5, 10 mg/kg) had no effect (Figure 3B,E,F).

### 2.6. Effect of LSE on SOD Activity in the Aorta and Serum in L-NAME Rats

**Figure 4:**
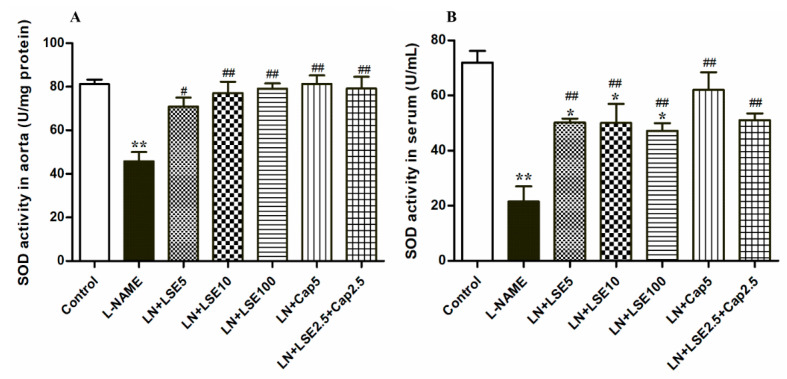
Effects of LSE and captopril on SOD activities in (**A**) aorta and (**B**) serum in L-NAME-induced hypertensive rats. Values are expressed as mean + SEM (*n* = 6). * *p* < 0.05, ** *p* < 0.01 versus control, ^#^ *p* < 0.05, ^##^ *p* < 0.01 versus L-NAME. CAP, captopril; L-NAME, LN, N^ω^-nitro-L-arginine methyl ester; LSE, lotus seed extract; SOD, superoxide dismutase.

L-NAME-induced hypertensive rats exhibited decreased SOD activity in both aorta and serum (*p* < 0.01 vs. control, Figure 4). Treatment with LSE (5, 10, and 100 mg/kg), captopril (5 mg/kg), or their combination (2.5 mg/kg each) elevated SOD activity (*p* < 0.05, 0.01 vs. L-NAME, Figure 4A,B).

### 2.7. Effects of LSE on Aortic eNOS Protein Expression and Plasma NO Concentration in L-NAME Rats

**Figure 5:**
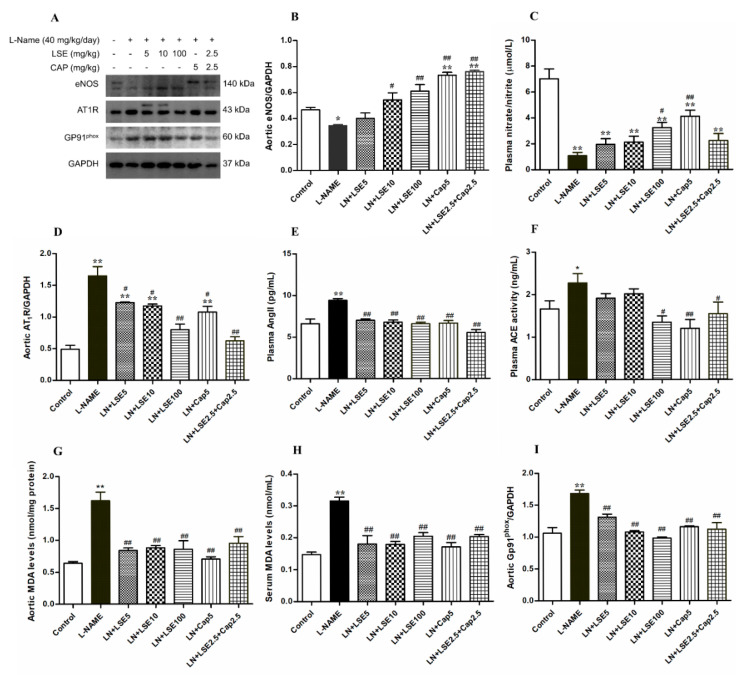
Effects of LSE and captopril on NO metabolites, RAS production, and oxidative stress markers in L-NAME-induced hypertensive rats. (**A**) Representative bands of the eNOS, AT_1_R, and Gp91^phox^ expression in aortic tissues, as investigated by western blot analysis. The quantitative results of the expression of aortic eNOS (**B**), plasma nitrate/nitrite (**C**), aortic AT_1_R (**D**), plasma Ang II (**E**), ACE activity (**F**), aortic MDA (**G**), serum MDA (**H**), Gp91^phox^ (**I**), in each group. Values are expressed as mean ± SEM. (*n* = 4–6). * *p* < 0.05, ** *p* < 0.01 versus control, ^#^ *p* < 0.05, ^##^ *p* < 0.01 versus L-NAME. ACE, angiotensin converting enzyme; Ang II, angiotensin II; CAP, captopril; L-NAME, LN, N^ω^-nitro-L-arginine methyl ester; LSE, lotus seed extract; MDA, malondialdehyde.

The aortic eNOS protein expression and plasma NO level were reduced in the L-NAME group (*p* < 0.05 vs. control, Figure 5A,B). LSE (10 and 100 mg/kg), captopril (5 mg/kg), and their combination (2.5 mg/kg each) significantly restored eNOS expression (*p* < 0.05, 0.01 vs. L-NAME, Figure 5B), while LSE (100 mg/kg) and captopril (5 mg/kg) also increase plasma NO level (*p* < 0.05, 0.01 vs. L-NAME, Figure 5C).

### 2.8. Effects of LSE on AT_1_R Protein Expressions, Plasma Ang II, and ACE Activity in L-NAME Rats

L-NAME-treated rats showed increased AT_1_R protein expression in aortic tissues and higher plasma Ang II levels compared to controls (*p* < 0.05, 0.01, Figure 5D,E). Treatments with LSE (5, 10, and 100 mg/kg), captopril (5 mg/kg), or their combination (2.5 mg/kg each) effectively suppressed AT_1_R overexpression and restored Ang II levels back to the control level. Additionally, ACE activity was significantly increased in L-NAME hypertensive rats compared to controls (*p* < 0.05, Figure 5F), which was significantly reduced by treatment with LSE (100 mg/kg), captopril (5 mg/kg) or their combination (2.5 mg/kg each) (*p* < 0.05, 0.01 vs. L-NAME, Figure 5F).

### 2.9. Effects of LSE on Aortic and Serum MDA and Aortic gp91^phox^ Protein Expressions

L-NAME treatment increased oxidative stress, as evidenced by elevated aortic and serum malondialdehyde (MDA) (*p* < 0.01 vs. control, Figure 5G,H). This elevation was accompanied by upregulated gp91^phox^ protein expression in hypertensive rats (Figure 5I). Treatment with LSE (5, 10, 100 mg/kg), captopril (5 mg/kg), or their combination (2.5 mg/kg each) normalized aortic and serum MDA to control level (Figure 5G,H) and significantly reduced gp91^phox^ expression (*p* < 0.01 vs. L-NAME, Figure 5I).

## 3. Discussion

This study demonstrated that LSE exerts protective effects against hemodynamic disturbances, cardiovascular remodeling, oxidative stress, RAS activation, and NO inactivation in L-NAME-induced hypertensive rats. These effects are mediated through upregulation of eNOS and enhanced NO bioavailability. LSE also reduced serum and tissue oxidative stress markers (MDA) and increased the activity of antioxidant enzymes (SOD). These changes were associated with the reduced gp91^phox^ protein expression in vascular tissues. Additionally, LSE decreased plasma Ang II levels, reduced ACE activity, and suppressed AT_1_R protein expression in hypertensive rats. Captopril, an ACE inhibitor used as a positive control, effectively lowered BP and alleviated these deleterious effects to normal levels. Notably, the combination of low-dose LSE (2.5 mg/kg) with half-dose captopril (2.5 mg/kg) produced greater antihypertensive effects than LSE alone, with efficacy comparable to the full dose of captopril, suggesting a potential additive or interactive effect in this hypertensive model.

The beneficial effects of LSE are likely attributed to the additive and/or interactive actions of its various phytochemical constituents. LC-MS analysis in this study identified several bioactive compounds in LSE, including alkaloids (nuciferine, neferine, armepavine, and isoliensinine) and flavonoids (catechin, kaempferol, apigenin, and rutin) (Table S1). Previous studies have shown that bisbenzylisoquinoline alkaloids present in LSE, such as isoliensinine and neferine, exhibit anticancer, antioxidant, anti-inflammatory, and antihypertensive properties [24,25]. Specifically, neferine has been shown to reduce SBP in L-NAME-induced hypertensive rats and to induce vasorelaxation via modulation of eNOS/NO/soluble guanylyl cyclase (sGC) pathway and through Ca^2+^ antagonism in isolated rat thoracic aorta [22]. Similarly, the flavonoid kaempferol, also present in LSE, has been reported to exert antihypertensive, cardioprotective, antioxidant, and anti-inflammatory effects in NO-dependent hypertensive rats, by suppressing the TNF-α pathway [26]. These findings support the hypothesis that the bioactive constituents of LSE contribute to its efficacy in alleviating the pathology of hypertension.

The primary pathology observed in hypertensive rats induced by L-NAME, a non-specific inhibitor of NO synthase (NOS), the enzyme responsible for producing NO, is a reduction in NO synthesis and availability. This impairment leads to endothelial dysfunction and vasoconstriction, resulting in increased total peripheral resistance (TPR) and development of hypertension [4]. Our study confirmed these findings, demonstrating that five weeks of L-NAME administration significantly elevated BP and HR, along with reduced NO production, as indicated by decreased levels of NO metabolites and eNOS protein expression in vascular tissues. These findings align with previous reports showing that L-NAME induces hypertension in rats [4,7]. Treatment with LSE effectively reduced BP and HR, associated with increased NO metabolites and eNOS expression, indicating improved NO bioavailability. These findings are consistent with previous studies on other plant extracts, such as *Carthamus tinctorius* and *Syzygium gratum*, which demonstrated similar antihypertensive effects in L-NAME–induced hypertensive rats. These extracts were also associated with elevated eNOS expression, improved NO availability, and modulation of oxidative stress and RAS activity [4,10].

It is well established that L-NAME induces eNOS uncoupling, leading to oxidative stress through increased production of superoxide anion (O_2_^−^) relative to NO [27,28]. This elevated vascular O_2_^−^ generation is associated with upregulation of the gp91^phox^ NADPH oxidase subunit expression in aortic tissues [6]. Our findings are consistent with these observations, showing that L-NAME-treated rats exhibited increased oxidative stress, as indicated by elevated serum and aortic MDA levels, decreased serum SOD activity, and upregulation of gp91^phox^ protein expression. Oxidative stress plays a critical role in the pathogenesis of hypertension and cardiovascular organ damage in L-NAME-induced hypertensive rats [29]. Histopathological changes in the LV and aorta, particularly increased wall thickness, may result not only from elevated afterload due to increased TPR, but also from the direct or indirect effects of excessive ROS [10,29]. This increased ROS can induce lipid peroxidation and simultaneously impair the endogenous antioxidant defence system [7]. Treatment with LSE improved antioxidant capacity and inhibited ROS production, which restored eNOS expression and NO metabolite levels, thereby alleviating cardiovascular remodeling in hypertensive rats. These findings are in line with previous study demonstrating that LSE mitigates reproductive damage by reducing ROS and enhancing antioxidant status in the testes and epididymis of L-NAME-induced hypertensive rats [23]. Substantial evidence indicates that flavonoids and alkaloids, the principal bioactive compounds of LSE, possess antioxidant [30,31] and antihypertensive properties [22,32]. Notably, flavonoids such as kaempferol alleviate hypertension and LV hypertrophy in L-NAME-induced hypertensive rats by reducing oxidative stress and inflammation, and increasing NO metabolites [30]. Therefore, the antioxidant activity of LSE likely contributes significantly to the attenuation of cardiovascular remodelling.

In the L-NAME-induced hypertension model, the RAS plays a critical role in hypertension development through the actions of Ang II, a potent vasoconstrictor that also enhances sympathetic nerve activity and salt and water retention [8]. Additionally, Ang II increases ROS production via AT_1_R activation, contributing to ventricular and vascular wall thickening [9,10]. This cascade further exacerbates endothelial dysfunction by promoting eNOS uncoupling and reducing NO bioavailability [33]. In the present study, the antihypertensive effects of LSE were associated with RAS inhibition, as evidenced by decreased plasma Ang II, ACE activity, and AT_1_R expression in L-NAME-induced hypertension following LSE treatment. These effects may be attributed, at least in part, to the flavonoids in LSE, which have been previously shown to exhibit ACE inhibitory activity [34].

Captopril, a commonly used ACE inhibitor, served as a positive control in this study and effectively reduced BP and vascular remodeling in L-NAME-induced hypertensive rats, consistent with previous findings [10]. Its therapeutic effects were associated with decreased oxidative stress, Ang II, ACE activity, and gp91^phox^ and AT_1_R expression, alongside increased NO levels, eNOS expression, and antioxidant activity [7]. Captopril (5 mg/kg) was more effective than LSE alone. However, due to its potential side effects, reducing the clinical dose is desirable [11]. Notably, a combination of low-dose LSE (2.5 mg/kg) with half-dose captopril (2.5 mg/kg) achieved comparable antihypertensive effects, suggesting a promising alternative therapeutic strategy. Nevertheless, these findings warrant confirmation in future clinical studies.

A limitation of the present study is the lack of pharmacokinetic evaluation of LSE, captopril, and their combination. As a result, it remains unclear whether the enhanced effects observed with the combination treatment are influenced by pharmacokinetic interactions. Additionally, the short treatment duration limits the ability to assess long-term efficacy and the potential for tolerance development. While the findings suggest promising antihypertensive effects of LSE, particularly when combined with captopril, their applicability to human hypertension remains to be established. Key factors such as oral bioavailability, safety, and therapeutic relevance in humans were not addressed in this study. Therefore, future investigations should include pharmacokinetic profiling, clinical trials, and prolonged administration of LSE to clarify its sustained antihypertensive potential and safety in the context of human hypertension.

## 4. Materials and Methods

### 4.1. Drugs and Chemicals

L-NAME and captopril were purchased from Sigma Chemical Co. (St. Louis, MO, USA). Hematoxylin and eosin (HandE) staining reagents were obtained from C.V. Laboratories Co. (Bangkok, Thailand). Formaldehyde, xylene, and absolute ethanol were supplied by RCI Labscan Ltd. (Bangkok, Thailand).

### 4.2. Plant Material and Preparation of Extract

An ethanolic extract of lotus seed was obtained from the Thailand Institute of Scientific and Technological Research in Bangkok, Thailand. The plant was authenticated by the Plant Variety Protection Department of Agriculture in Bangkok, Thailand. The voucher specimen (BK No. 082574) was deposited in the herbarium of the Bangkok Herbarium, Bangkok, Thailand. The LSE was prepared as previously described [23]. Briefly, fresh lotus seeds were dried at 50 °C for 24 h and ground into a coarse powder. The powder was extracted with 95% ethanol via sonication for 30 min, and the residue was filtered using Whatman filter paper No. 1. This extraction was repeated twice, and the supernatants were combined. The final extract yield was 3.82 ± 0.56% (*w/w*) of the seed’s dry weight. The extract was then frozen and stored at −20 °C for future studies.

### 4.3. HPLC-ESI-QTOF-MS/MS Analysis

The structure elucidation of chemical constituents in LSE was performed using an Agilent 1260 Infinity Series (Agilent, Waldbronn, Germany) coupled with an Agilent 6540 Q-TOF-MS spectrometer (Agilent Technologies, Singapore). Sample volume 10 µL at concentration 20 mg/mL was flowed to the Luna C18(2) column size 150 × 4.6 mm, 5 µm (Phenomenex, CA, USA). The mobile phase constant flow is at 0.5 mL /min. The binary gradient elution system was composed of water type1 (Millipore, MA, USA) as solvent A and acetonitrile as solvent B, and both contained 0.1% of formic acid (*v/v*). The linear gradient elution was 5–95% for solvent B in 0- 30 min, hold at 95% (*v/v*) B for 10 min, and post-run for 5 min. The column temperature was set at 35 °C. The HPLC instrument was coupled to an electrospray ionization (ESI) source and a proprietary Agilent dual nebulizer. The MS detection was operated under the following conditions: drying gas (N2) flow rate, 10.0 L/min; drying gas temperature, 350 °C; nebulizer pressure 30 psig; capillary 3500 V; skimmer 65 V; octapole RFV 750 V; and fragmentor voltage 250 V in negative mode and 100 V in positive mode. The mass range was configured from *m*/*z* 100 to 1000 Da. For untargeted MS/MS acquisition, collision energies of 10, 20, and 40 V were applied. Data acquisition and analysis were managed using Agilent MassHunter Acquisition Software Version B.05.01 and Qualitative Analysis Software B 06.0 (Agilent Technologies, Santa Clara, CA, USA). Accurate mass measurements were determined. The errors between the measurement and theoretical mass of less than 5 ppm are acceptable.

### 4.4. Animals and Experimental Protocols

Fifty-six male Sprague Dawley rats (6 weeks old, weighing 200–220 g) were purchased from Nomura Siam International Co., Ltd. (Bangkok, Thailand). The animals were housed in groups of 2–3 per cage at the Naresuan University Center for Animal Research (NUCAR) under controlled environmental conditions, including a 12-h light-dark cycle and a maintained temperature of 22 ± 1 °C. All rats had free access to a standard rodent diet and tap water. All experimental procedures were conducted in accordance with institutional guidelines for the care and use of laboratory animals and were approved by the Naresuan University Animal Care and Use Committee (NUACUC), protocol number NU-AE 630607. After a one-week acclimatization period, the rats were randomly assigned to one normotensive control group and six hypertensive groups (*n* = 8 per group). The normotensive control group was given distilled water via intragastric gavage daily, while hypertensive groups were treated with L-NAME (40 mg/kg/day) in their drinking water daily and concurrently administered with either distilled water, LSE (5, 10, or 100 mg/kg/day), captopril (5 mg/kg/day), or a combination of LSE (2.5 mg/kg/day) and captopril (2.5 mg/kg/day) via intragastric gavage for 5 weeks.

The normotensive control group received distilled water via intragastric gavage once daily. Hypertensive groups were treated with L-NAME (40 mg/kg/day) in their drinking water and concurrently administered either distilled water, LSE (5, 10, or 100 mg/kg/day), captopril (5 mg/kg/day), or a combination of LSE (2.5 mg/kg/day) and captopril (2.5 mg/kg/day) via intragastric gavage for 5 weeks. LSE and captopril were dissolved in distilled water and administered orally once daily at a volume of 1 mL/kg throughout the treatment period.

Captopril was used as a reference drug to evaluate its antihypertensive effect in L-NAME-induced hypertensive rats. The selected doses of LSE (5, 10, and 100 mg/kg), L-NAME (40 mg/kg), and captopril (5 mg/kg) were based on data from a previous study [23].

### 4.5. Blood Pressure Measurement

The systolic blood pressure (SBP) of all rats was measured weekly for five weeks using a non-invasive tail-cuff plethysmography method (NIBP, AD Instrument, Sydney, Australia). Conscious rats were placed in a restraining apparatus maintained at 32–35 °C, with their tails inserted into an automatically inflating and deflating cuff. For each rat, SBP was recorded as the average of three consecutive measurements taken at 15-min intervals. At the end of the experiment (week 5), hemodynamic parameters including SBP, diastolic blood pressure (DBP), mean arterial blood pressure (MAP), and heart rate (HR) were directly measured in anesthetized rats using canulation technique as previously described [35]. Briefly, rats were anesthetized with a 1% isoflurane and oxygen mixture, and hemodynamic values were recorded via catheterization of the common carotid artery. Measurements were continuously monitored for 15 min using LabChart 8 software and analyzed offline using the LabChart physiological data analysis platform.

Systolic blood pressure (SBP) was measured weekly over a five-week period using a non-invasive tail-cuff plethysmography system (NIBP, AD Instruments, Sydney, Australia). Rats were placed in a temperature-controlled restraining apparatus (32–35 °C), and their tails were inserted into an automated inflatable cuff. SBP was calculated as the mean of three measurements obtained at 15-min intervals. At the end of the experimental period (week 5), hemodynamic parameters, including SBP, diastolic blood pressure (DBP), mean arterial pressure (MAP), and heart rate (HR), were directly assessed in anesthetized rats using a cannulation technique, as previously described.

### 4.6. Blood and Tissue Sample Collection

Following blood pressure measurement, rats were sacrificed under isoflurane anesthetized, and blood was collected from the carotid artery. The samples were centrifuged at 3000 rpm for 10 min to obtain the plasma and serum [36]. The heart, aorta, and liver were dissected, and any surrounding fat pads were removed prior to determining their absolute weights. Relative organ weights were calculated as per previously published study [37]. Subsequently, the heart and aorta were prepared for biochemical evaluations and histological analysis.

### 4.7. Histological and Morphometric Examination

Morphological changes in the left ventricle (LV) and abdominal aorta were evaluated. These tissues were fixed in 10% neutral buffered formalin and then underwent standard paraffin processing procedures, resulting in sections of 5 µm thickness. Following this, hypertrophy of the LV and histology of abdominal aorta were examined using hematoxylin and eosin (HandE) staining. The ratio of LV cross-sectional area (CSA) to luminal area was computed. The sections were observed and captured using a light microscope (BX53F2, Olympus, Tokyo, Japan). Morphological assessments were analyzed utilizing Image J software version 1.54 from the National Institute of Mental Health in Bethesda, Maryland, USA.

### 4.8. Assay of Oxidative Stress and Antioxidant Defense System

Lipid peroxidation levels in the serum and aorta were determined by measuring MDA using an MDA assay kit (MAK085, Sigma-Aldrich, Bayern, Germany), following the manufacturer’s instructions. The thoracic aorta was homogenized, incubated with thiobarbituric acid (TBA) for 60 min at 95 °C, and then rapidly cooled to −20 °C for 10 min. MDA formation was indicated by the development of a pink color, which was measured spectrophotometrically at 532 nm. MDA concentrations were expressed as nmol per mg of protein. Total protein content in tissue samples was quantified using the Bradford assay [38].

Superoxide dismutase (SOD) activity was measured using a commercial assay kit (Catalog No. 19160, Sigma-Aldrich, Bayern, Germany). The thoracic aorta was homogenized in 50 mM Tris-HCl buffer (pH 7.4) and centrifuged at 13,000× *g* for 10 min at 4 °C. The resulting supernatant was collected, and absorbance was measured at 450 nm to detect the yellow-colored product. SOD activity was calculated and expressed as units per milligram of protein (U/mg protein).

### 4.9. Assays of Plasma Angiotensin-Converting Enzyme (ACE) Activity

The ACE activity in the plasma was performed using the ACE assay kit (ab263889, Abcam, Cambridge, MA, USA), following the manufacturer’s instructions.

### 4.10. Assay of Plasma Angiotensin II Concentration

The concentration of plasma Ang II was measured using an Ang II ELISA kit (ab285306, Abcam, Cambridge, MA, USA). The procedure was carried out as per the manufacturer’s instructions.

### 4.11. Assay of Plasma NO Level

Nitrite and nitrate, stable end products of NO metabolism, can be used as indirect markers of NO presence. Plasma NO levels were measured using the colorimetric NO Kit (ab65328; Abcam Cambridge, MA, USA) following the manufacturer’s protocol. The method involves a two-step process: first, nitrate is converted to nitrite using nitrate reductase; second, nitrite is converted to a deep purple azo compound using the Griess reaction. The absorbance of this compound was measured at 540 nm with a microplate reader to determine the levels of nitrate/nitrite.

### 4.12. Expression of AT_1_R, gp91^phox^, and eNOS Proteins by Western Blotting Analysis

Total protein lysates were extracted from aortic tissues using radioimmunoprecipitation assay (RIPA) buffer (Tris-HCl [pH 7.5], NaCl, Triton X-100, sodium deoxycholate, EDTA, SDS, and sodium fluoride) supplemented with protease and phosphatase inhibitors (MilliporeSigma, Burlington, MA, USA; Thermo Fisher Scientific, Waltham, MA, USA). Protein concentrations were determined by the Bradford assay using serial dilutions of bovine serum albumin (BSA; Capricorn Scientific, Boston, MA, USA) ranging from 0 to 0.5 mg/mL as standards. Equal amounts of protein were separated via SDS-PAGE (5% stacking gel and 12–15% separating gel) and transferred onto polyvinylidene difluoride (PVDF) membranes (Bio-Rad Laboratories, Hercules, CA, USA). Membranes were blocked with 5% BSA and incubated overnight at 4 °C with the following primary antibodies: rabbit anti-GAPDH (MilliporeSigma, Burlington, MA, USA), rabbit anti-AT1R, rabbit anti-gp91^phox^, and rabbit anti-eNOS (Cell Signaling Technology, Danvers, MA, USA). After washing, membranes were incubated with HRP-conjugated goat anti-rabbit IgG (Cell Signaling Technology, Danvers, MA, USA) for 1 h at room temperature in the dark. Protein bands were visualized using enhanced chemiluminescence (ECL) reagents (Bio-Rad Laboratories, Hercules, CA, USA; MilliporeSigma, Burlington, MA, USA) and imaged with the ImageQuant™ LAS 500 system (Cytiva, Amersham, UK). Densitometric analysis was performed using ImageJ software version 1.54 (National Institutes of Health, Bethesda, MD, USA), and protein expression levels were normalized to GAPDH.

### 4.13. Statistical Analysis

All results are presented as mean ± standard error of the mean (SEM). Statistical analyses were performed using GraphPad Prism version 8.0 (GraphPad Software Inc. (San Diego, CA, USA). Group comparisons were conducted using one-way analysis of variance (ANOVA) followed by Tukey’s post hoc test. A *p*-value of less than 0.05 was considered statistically significant.

## 5. Conclusions

This study is the first to demonstrate that LSE exerts antihypertensive effects through mechanisms involving the reduction of vascular remodeling, enhancement of antioxidant defenses, inhibition of the RAS, and improved NO bioavailability. Notably, the combination of low-dose LSE with captopril produced greater therapeutic effects than LSE alone and was comparable in efficacy to captopril. These findings support the potential of LSE as a complementary approach to conventional antihypertensive therapy. Further research is warranted to elucidate the underlying molecular mechanisms and to evaluate its long-term safety and clinical applicability.

## Figures and Tables

**Table 1:** Effect of LSE and captopril on arterial blood pressure, heart, body weight, heart weight, and liver weight in normal and L-NAME-induced hypertensive rats.

Parameters							
Groups	SBP (mmHg)	DBP (mmHg)	MAP (mmHg)	HR (bpm)	BW (g)	HW/BW (per BW%)	LW/BW (per BW%)
**Control**	116.4 ± 3.3	81.3 ± 2.7	93.0 ± 2.8	349.4 ± 8.4	534.6 ± 9.2	0.28 ± 0.01	3.2 ± 0.2
**L-NAME**	172.7 ± 5.1 **	129.3 ± 3.5 **	143.8 ± 3.6 **	388.1 ± 12.9 *	533.1 ± 17.6	0.26 ± 0.01	3.3 ± 0.2
**LN + LSE5**	148.6 ± 7.6 **^#^	103.3 ± 4.2 *^##^	117.8 ± 5.3 *^##^	321.1 ± 5.0 ^#^	565.9 ± 11.2	0.25 ± 0.01	3.2 ± 0.1
**LN + LSE10**	145.7 ± 9.3 **^#^	108.6 ± 7.7 **^#^	120.5 ± 8.1 **^#^	326.4 ± 10.9 ^#^	548.4 ± 9.2	0.27 ± 0.01	3.3 ± 0.1
**LN + LSE100**	145.4 ± 8.3 **^#^	108.7 ± 7.5 **^#^	121.0 ± 7.7 **^#^	333.2 ± 10.5 ^#^	537.8 ± 9.8	0.28 ± 0.01	3.2 ± 0.1
**LN + CAP5**	127.0 ± 4.6 ^##^	93.6 ± 3.0 ^##^	104.7 ± 3.5 ^##^	335.4 ± 5.0 ^#^	528.6 ± 7.6	0.25 ± 0.01	3.5 ± 0.1
**LN + LSE2.5**	126.0 ± 2.9 ^##^	91.9 ± 3.2 ^##^	103.3 ± 3.0 ^##^	320.4 ± 8.3 ^#^	568.1 ± 9.1	0.25 ± 0.01	3.5 ± 0.1
**+Cap2.5**							

* *p* < 0.05, ** *p* < 0.01 versus control, ^#^ *p* < 0.05, ^##^ *p* < 0.01 versus L-NAME. BW, body weight; CAP, captopril; DBP, diastolic blood pressure; HR, heart rate; HW, heart weight; L-NAME, LN, N^ω^-nitro-L-arginine methyl ester; LW, liver weight; MAP, mean arterial pressure; LSE, lotus seed extract; SBP, systolic blood pressure.

## Data Availability

Data are available from the corresponding authors upon reasonable request.

## Supplementary Materials

The following supporting information can be downloaded at: https://www.mdpi.com/article/10.3390/ph18081156/s1, Figure S1. Representative bands of eNOS, AT_1_R, and GP91^phox^. Table S1: Alkaloids and flavonoids found in lotus seed ethanolic extract purposed using LC-ESI-QTOF. Table S2: Raw data from four independent western blot experiments showing the expression levels of AT_1_R, gp91 ^phox^, and eNOS proteins.

## Abbreviations

The following abbreviations are used in this manuscript:

ACE	Angiotensin converting enzyme
Ang II	Angiotensin II
BP	Blood pressure
Cap	Captopril
DBP	Diastolic blood pressure
eNOS	Endothelial nitric oxide synthase
HR	Heart rate
HandE	Hematoxylin and eosin
L-NAME	N^ω^-nitro-L-arginine methyl ester
LV	Left ventricular
LSE	Lotus seed extract
MAP	Mean arterial pressure
MDA	Malondialdehyde
NO	Nitric oxide
NOS	Nitric oxide synthase
O_2_^−^	Superoxide anion
RAS	Renin-angiotensin system
ROS	Reactive oxygen species
SBP	Systolic blood pressures
