# Supplementary material for: Antihypertensive Effects of Lotus Seed (Nelumbo nucifera Gaertn.) Extract via eNOS Upregulation and Oxidative Stress Reduction in L-NAME-Induced Hypertensive Rats
Source: Pharmaceuticals (Basel). 2025 Aug 4;18(8):1156. doi: 10.3390/ph18081156 (PMC12389499; doi:10.3390/ph18081156)
Supplement: Supplementary file 1 [file pharmaceuticals-18-01156-s001.zip › pharmaceuticals-3756881 Figure S1.pdf]

Figure S1. : Representative bands of eNOS, AT1R and GP91^{phox}

L-Name (40 mg/kg/day)	-	+	+	+	+	+	+
LSE (mg/kg)	-	-	5	10	100	-	5
CAP (mg/kg)	-	-	-	-	-	5	5

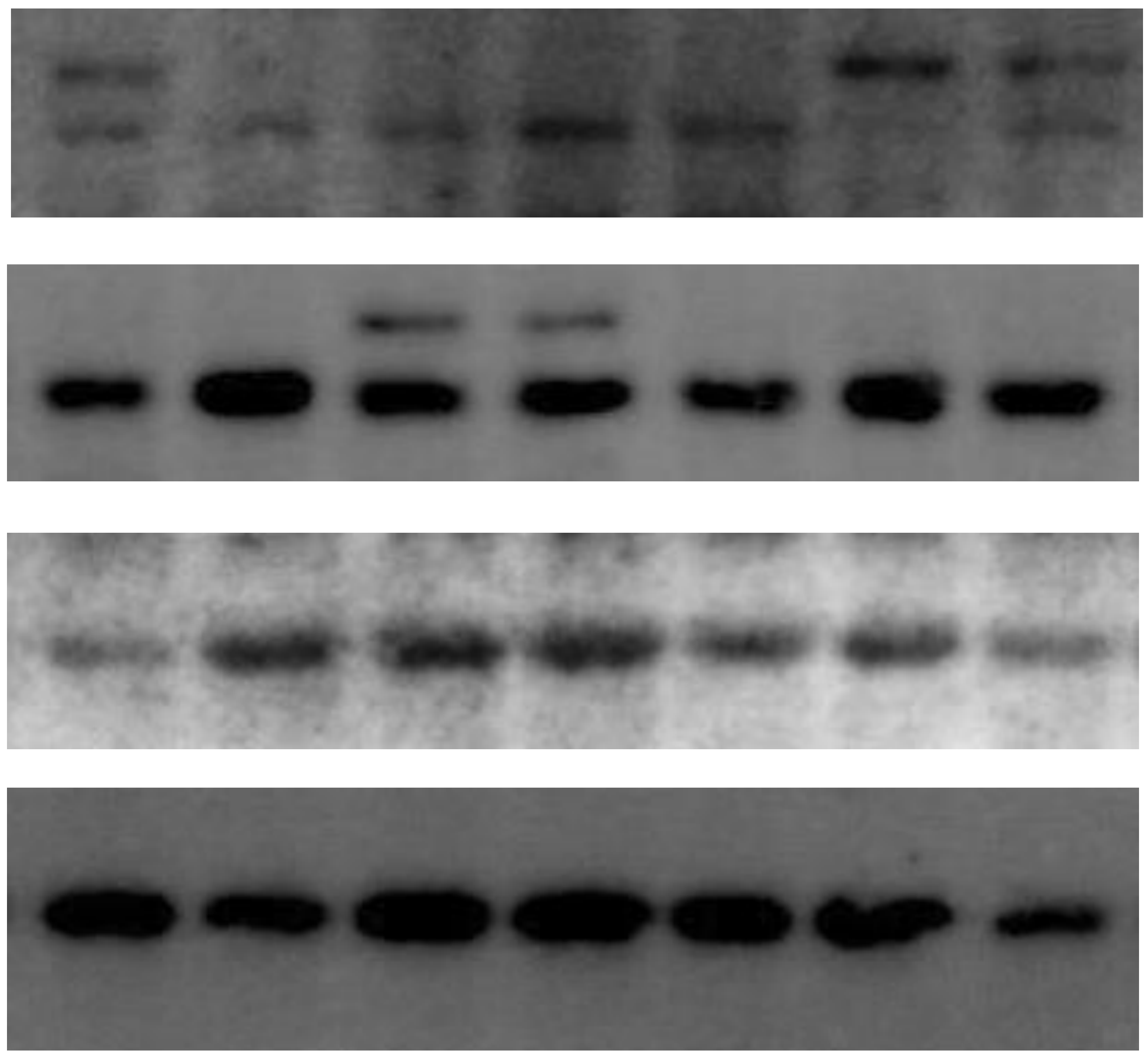

eNOS 140 kDa

AT1R 43 kDa

GP91^{phox} 60 kDa

GAPDH 37 kDa

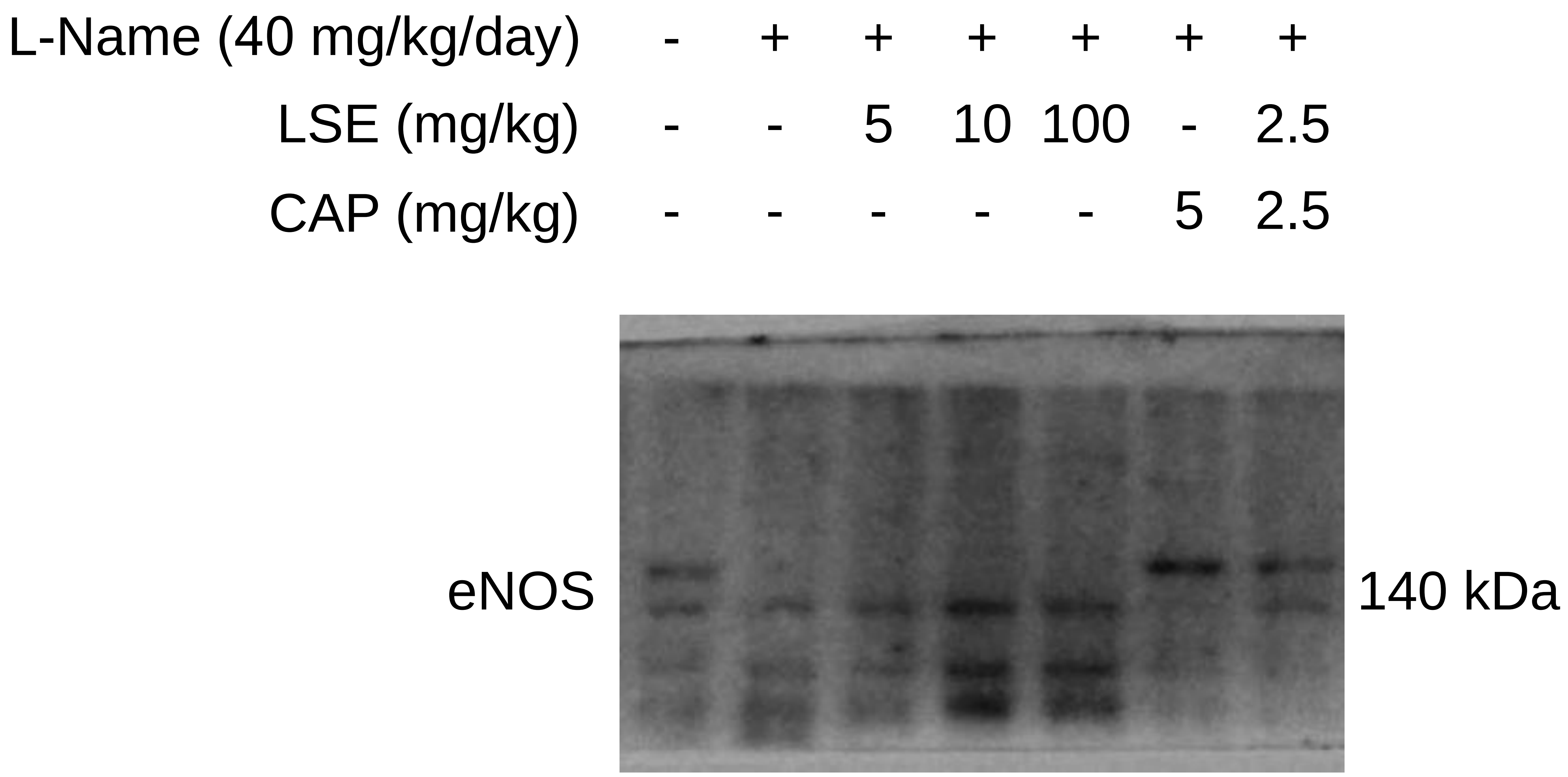
L-Name (40 mg/kg/day) - + + + + + +
LSE (mg/kg) - - 5 10 100 - 2.5
CAP (mg/kg) - - - - - 5 2.5
eNOS
140 kDa

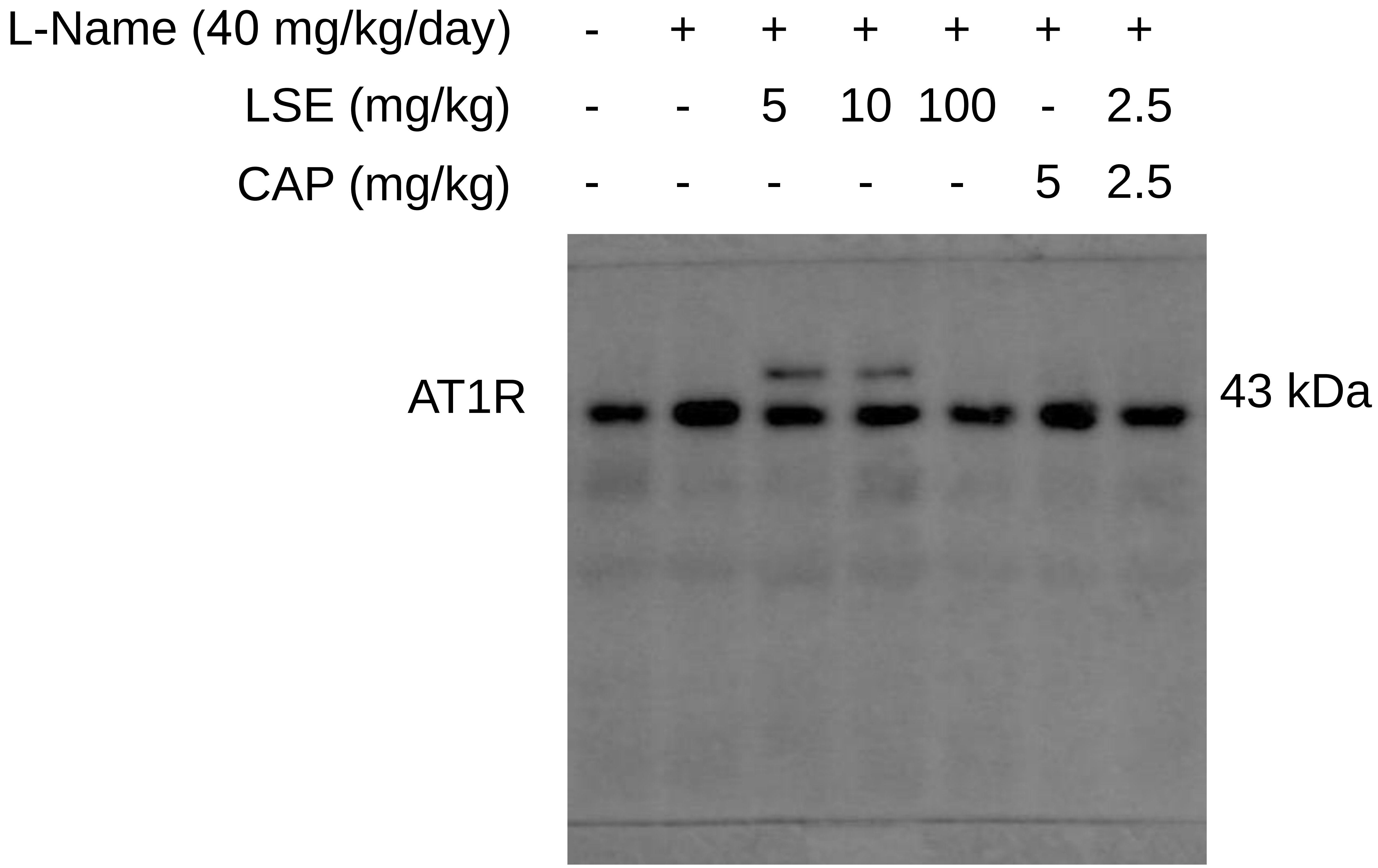
L-Name (40 mg/kg/day) - + + + + + +
LSE (mg/kg) - - 5 10 100 - 2.5
CAP (mg/kg) - - - - - 5 2.5
AT1R
43 kDa

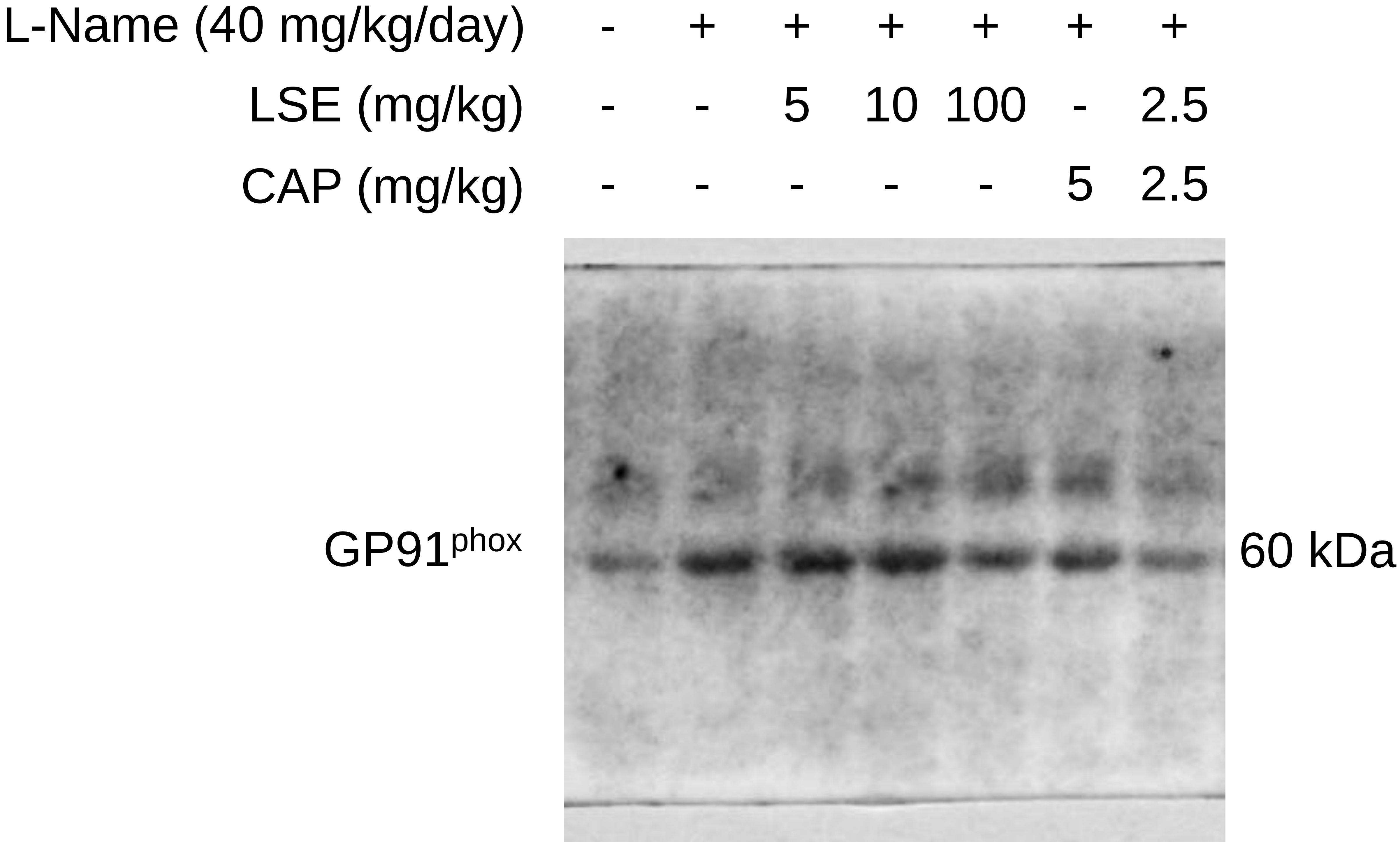

L-Name (40 mg/kg/day) - + + + + + +
LSE (mg/kg) - - 5 10 100 - 2.5
CAP (mg/kg) - - - - - 5 2.5
GP91phox
60 kDa

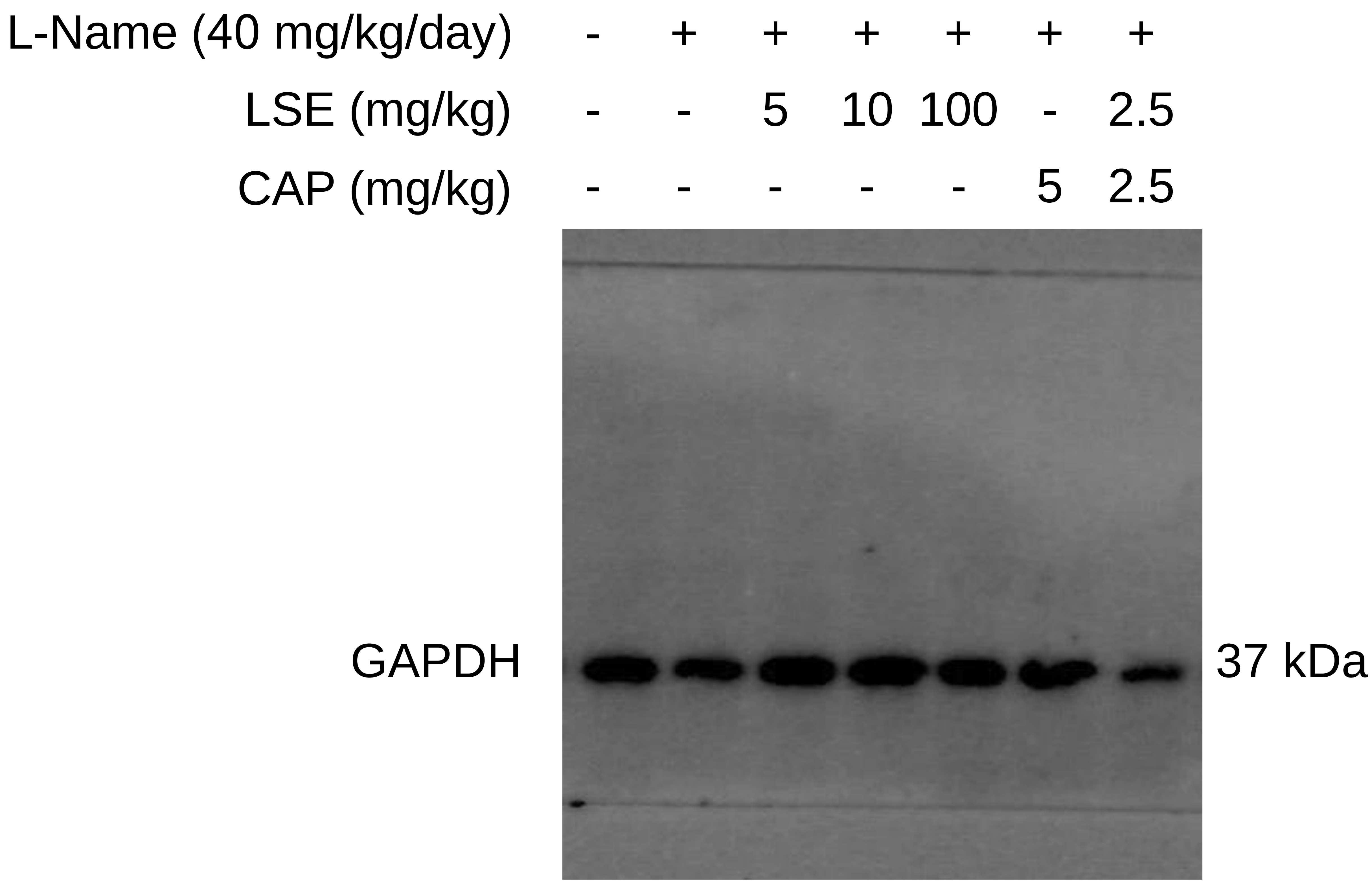
L-Name (40 mg/kg/day) - + + + + + +
LSE (mg/kg) - - 5 10 100 - 2.5
CAP (mg/kg) - - - - - 5 2.5
GAPDH
37 kDa
