# Supplementary material for: Antihypertensive Effects of Lotus Seed (Nelumbo nucifera Gaertn.) Extract via eNOS Upregulation and Oxidative Stress Reduction in L-NAME-Induced Hypertensive Rats
Source: Pharmaceuticals (Basel). 2025 Aug 4;18(8):1156. doi: 10.3390/ph18081156 (PMC12389499; doi:10.3390/ph18081156)
Supplement: Supplementary file 1 [file pharmaceuticals-18-01156-s001.zip › pharmaceuticals-3756881 Table S1.pdf]

Table S1 Alkaloids and flavonoids found in lotus seed ethanolic extract purposed using LC-ESI-QTOF

No.	RT (min)	m/z	Adduct	MS/MS	Tentative identification	Formula	Error (ppm)
Alkaloids							
1	3.071	328.1029	[M+H]+	*	Blepharin	C14H17NO8	-0.63
2	7.987	272.128	[M+H]+	255.1016,161.0592, 123.0425,107.0489,55.0181	Demethylcoclaurine	C16H17NO3	0.44
3	8.126	286.1436	[M+H]+	225.0901,209.0944, 161.0593,143.0488, 123.0437,107.0488,91.0534, 77.0381	Coclaurine	C17H19NO3	0.59
4	8.126	314.1752	[M+H]+	269.1173,237.0910,209.0960, 192.1018,137.0590,107.0488, 77.0385,58.0649	Armepavine	C19H23NO3	-0.41
5	8.531	299.152	[M+2H]+	283.6295,252.1148,192.1017, 175.0754,143.0494,107.0486	Nelumboferine	C36H40N2O6	-1.15
		597.2958	[M+H]+				
6	8.643	314.1748	[M+H]+	269.1173,237.0902,107.0487, 77.0378,58.0648	Armepavine	C19H23NO3	-0.41
7	9.17	300.1595	[M+H]+	269.1169,237.0909,175.0752, 145.0642,107.0487,91.0533, 77.0385	N-methyl-coclaurine	C18H21NO3	-0.27
8	9.38	300.1594	[M+H]+	269.1171,237.0906,209.0957, 175.0750,107.0487,77.0376	N-methyl-Isococlaurine	C18H21NO3	0.07
9	9.463	299.152	[M+2H]+	192.1032,107.0475	Nelumborine	C36H40N2O6	-1.28
10	9.562	312.1596	[M+H]+	269.1172,238.0976,206.1174, 177.0892,121.0670,85.0278	Pronuciferine	C19H21NO3	-0.58
11	9.638	306.1596	[M+2H]+	266.1298,192.1014,175.0749, 158.0721,121.0643	Isoliensinine	C37H42N2O6	-1
		611.3127	[M+H]+	537.2290,475.2231,419.2073, 192.1015,121.0635			
12	9.803	328.1909	[M+H]+	283.1317,252.1136,228.1548, 207.1236.189.0898,165.0895, 145.0490,107.0483,85.0278, 58.0647	L-O-Methylarmepavine	C20H25NO3	-0.55
13	10.313	313.1674	[M+2H]+	189.0905,107.0487,58.0648	Neferine	C38H44N2O6	0.38
		625.3286	[M+H]+	489.2373,206.1176		C38H44N2O6	-2.22
14	10.414	314.175	[M+H]+	192.1005,121.0643,60.0438	Armepavine	C19H23NO3	0.22
15	10.882	286.1438	[M+H]+	*	Coclaurine	C17H19NO3	-0.11

16	10.904	300.1595	[M+H]+	*	4'-N-Methyl-coclaurine	C18H21NO3	-0.27
17	11.189	268.1333	[M+H]+	251.1065,219.0800,191.0849,165.0686,	Asimilobine	C17H17NO2	-0.35
18	11.47	282.1498	[M+H]+	251.1063,219.0803,191.0855,165.0692	Methyl asimilobine	C18H19NO2	-3.35
19	13.005	282.1483	[M+H]+	265.1225,234.1031,	Methyl asimilobine	C18H19NO2	1.97
20	13.254	296.1643	[M+H]+	265.1223,234.1029,	Nuciferine	C19H21NO2	0.69
21	15.55	144.0454	[M-H]-	115.0407,65.9985	4-Hydroxyquinoline	C9H7NO	0.61
Flavonoids							
1	5.012	633.2158	[M-H]-	*	Uvarinol	C36H30O7	-2.96
2	7.235	325.1081	[M-H]-	164.0699,124.0060,101.0231,79.9567,59.0129	omega-Hydroxy moracin N	C19H18O5	0.15
3	9.977	593.1522	[M-H]-	473.1021,413.0825,383.0711,353.0605	Apigenin-6,8-di-C-glycopyranoside (Vicenin-2)	C27H30O15	-1.7
		595.1669	[M+H]+	457.1155,379.0806,307.0606		C27H30O15	-1.94
4	10.164	289.0724	[M-H]-	245.0789,205.0474,123.0431,109.0279	Catechin	C15H14O6	-2.21
5	10.456	563.1414	[M-H]-	503..1126,473.1029,443.0923,383.0725,353.0672	Isomollupentin 4'-O-glucoside	C26H28O14	-1.37
6	10.638	565.1563	[M+H]+	529.1355,427.1019,379.0808,349.0710,325.0690, 295.0592	Apigenin 6-C-glucoside 8-C-arabinoside (Schaftoside)	C26H28O14	-1.98
		563.1415	[M-H]-	353.0617,161.0796,89.0235,59.0122	Apigenin 6-C-glucosyl-8-C-arabinoside (Schaftoside)	C26H28O14	-1.55
7	10.91	563.1416	[M-H]-	503.1109,473.1026,443.0925,413.0832,383.0725,353.0623	Apigenin 6-C-arabinoside 8-C-glucoside (Isoschaftoside)	C26H28O14	-1.72
		565.1561	[M+H]+	529.1334,427.1018,325.0698		C26H28O14	-1.55
8	10.874	449.1033	[M-H]-	287.0542,269.0425,259.0581	Eriodictyol 7-O-glucoside	C21H22O11	12.55
9	11.016	447.0938	[M-H]-	*	Luteolin 6-C-	C21H20O11	-1.15

					glucoside (Isorientin)		
10	11.403	483.0703	[M-H]-	*	Kaempferol 7-O-glucoside	C21H20O11	-1.15
11	11.579	577.1567	[M-H]-	457.1079,353.0624	2"-O-alpha-L-Rhamnosyl-6-fucosyl-luteolin	C27H30O14	-0.73
12	11.799	609.1462	[M-H]-	301.0315,125.0206	Rutin	C27H30O16	-0.15
13	12.048	593.1429	[M-H]-	285.0353,151.0011,125.0246	Kaempferol-3-O-robinobioside	C27 H30 O15	13.95
14	12.112	431.0989	[M-H]-	311.0509,283.0574	Apigenin 8-C-glucoside	C21H20O10	-1.23

***MS/MS are not achievement**
