# Supplementary material for: Antihypertensive Effects of Lotus Seed (Nelumbo nucifera Gaertn.) Extract via eNOS Upregulation and Oxidative Stress Reduction in L-NAME-Induced Hypertensive Rats
Source: Pharmaceuticals (Basel). 2025 Aug 4;18(8):1156. doi: 10.3390/ph18081156 (PMC12389499; doi:10.3390/ph18081156)
Supplement: Supplementary file 1 [file pharmaceuticals-18-01156-s001.zip › pharmaceuticals-3756881 Table S2.pdf]

Raw intensities

eNOS	CON	LN	LN+LSE5	LN+LSE10	LN+LSE100	LN+CAP5	LN+CAP5+LSE5
N1	1945.891	1316.548	2147.468	2889.012	2780.548	3100.619	2763.912
N2	2026.362	1286.660	1496.656	2026.068	3063.602	3431.632	3028.963
N3	2002.351	1203.562	1632.561	2691.893	2628.862	3000.688	2960.100
Mean	1991.535	1268.923	1758.895	2535.658	2824.337	3177.646	2917.658
SD	41.312	58.544	343.306	452.189	220.653	225.562	137.528

gp91 p	CON	LN	LN+LSE5	LN+LSE10	LN+LSE100	LN+CAP5	LN+CAP5+LSE5
N1	4031.477	5871.602	5448.598	4869.841	4155.134	4958.477	4845.305
N2	5064.620	6260.644	5930.632	5016.624	4631.321	5026.634	3961.118
N3	5196.632	6403.477	5880.001	5210.100	4926.364	5102.604	4030.005
Mean	4764.243	6178.574	5753.077	5032.188	4570.940	5029.238	4278.809
SD	638.017	275.271	264.899	170.663	389.144	72.099	491.807

AT1R	CON	LN	LN+LSE5	LN+LSE10	LN+LSE100	LN+CAP5	LN+CAP5+LSE5
N1	2708.770	7047.518	5330.305	5660.589	4022.004	4898.598	2193.912
N2	2107.621	6062.420	5252.612	5206.019	2860.527	5226.825	3006.020
N3	1806.986	5054.632	5561.323	5589.008	4264.953	3862.481	1982.236
Mean	2207.792	6054.857	5381.413	5485.205	3715.828	4662.635	2394.056
SD	459.162	996.465	160.576	244.417	750.607	712.122	540.441

GAPDI	CON	LN	LN+LSE5	LN+LSE10	LN+LSE100	LN+CAP5	LN+CAP5+LSE5
N1	4557.305	3688.355	4437.719	4667.891	4316.719	4238.276	3679.648
N2	4261.628	3726.636	4257.531	4654.166	4554.985	4457.826	4067.682
N3	4702.615	3602.538	4489.632	4702.705	5140.623	4348.560	3800.534
Mean	4507.183	3672.510	4394.961	4674.921	4670.776	4348.221	3849.288
SD	224.726	63.548	121.815	25.021	423.981	109.775	198.558

Normalized with GAPDH

eNOS	CON	LN	LN+LSE5	LN+LSE10	LN+LSE100	LN+CAP5	LN+CAP5+LSE5
N1	0.427	0.357	0.484	0.619	0.644	0.732	0.751
N2	0.475	0.345	0.352	0.435	0.673	0.770	0.745
N3	0.426	0.334	0.364	0.572	0.511	0.690	0.779
Mean	0.443	0.345	0.400	0.542	0.609	0.730	0.758
SD	0.028	0.011	0.073	0.095	0.086	0.040	0.018

gp91 p	CON	LN	LN+LSE5	LN+LSE10	LN+LSE100	LN+CAP5	LN+CAP5+LSE5
N1	0.885	1.592	1.228	1.043	0.963	1.170	1.317
N2	1.188	1.680	1.393	1.078	1.017	1.128	0.974
N3	1.105	1.777	1.310	1.108	0.958	1.173	1.060
Mean	1.059	1.683	1.310	1.076	0.979	1.157	1.117
SD	0.157	0.093	0.083	0.032	0.033	0.026	0.178

AT1R	CON	LN	LN+LSE5	LN+LSE10	LN+LSE100	LN+CAP5	LN+CAP5+LSE5
N1	0.594	1.911	1.201	1.213	0.932	1.156	0.596
N2	0.495	1.627	1.234	1.119	0.628	1.173	0.739
N3	0.384	1.403	1.239	1.188	0.830	0.888	0.522
Mean	0.491	1.647	1.225	1.173	0.796	1.072	0.619
SD	0.105	0.254	0.020	0.049	0.155	0.160	0.110

Normalized with CONTROL

eNOS	CON	LN	LN+LSE5	LN+LSE10	LN+LSE100	LN+CAP5	LN+CAP5+LSE5
N1	1.000	0.836	1.133	1.449	1.509	1.713	1.759
N2	1.001	0.727	0.740	0.916	1.416	1.621	1.568
N3	1.000	0.784	0.854	1.344	1.200	1.620	1.828
Mean	1.000	0.782	0.909	1.237	1.375	1.651	1.718
SD	0.001	0.055	0.202	0.282	0.158	0.054	0.135

gp91 p	CON	LN	LN+LSE5	LN+LSE10	LN+LSE100	LN+CAP5	LN+CAP5+LSE5
N1	1.000	1.799	1.387	1.179	1.088	1.322	1.488
N2	1.000	1.414	1.173	0.907	0.856	0.949	0.820
N3	1.000	1.609	1.185	1.003	0.867	1.062	0.960
Mean	1.000	1.607	1.248	1.030	0.937	1.111	1.089
SD	0.000	0.192	0.121	0.138	0.131	0.191	0.352

AT1R	CON	LN	LN+LSE5	LN+LSE10	LN+LSE100	LN+CAP5	LN+CAP5+LSE5
N1	1.001	3.217	2.022	2.042	1.569	1.946	1.004
N2	0.999	3.286	2.492	2.260	1.269	2.369	1.493
N3	1.001	3.654	3.226	3.095	2.161	2.313	1.358
Mean	1.000	3.386	2.580	2.465	1.666	2.209	1.285
SD	0.001	0.235	0.607	0.556	0.454	0.230	0.253
